# Generation of tumour-specific cytotoxic T-cell clones from histocompatibility leucocyte antigen-identical siblings of patients with melanoma

**DOI:** 10.1038/sj.bjc.6603243

**Published:** 2006-07-04

**Authors:** D J Gottlieb, Y-C Li, I Lionello, S Tanzarella, M Marangolo, K F Bradstock, V Russo, C Traversari

**Affiliations:** 1 Leukaemia Research Laboratory, University of Sydney, Westmead Hospital, Sydney NSW 2145, Australia; 2 Molmed, Via Olgettina 58, Milan 20132, Italy; 3 Cancer Immunotherapy and Gene Therapy Program, Cancer Gene Therapy Unit, Scientific Institute H San Raffaele, Via Olgettina 58, Milan 20132, Italy

**Keywords:** human, tumour immunity, T cells, transplantation, melanoma

## Abstract

Lymphodepletion and infusion of autologous expanded tumour-infiltrating lymphocytes is effective therapy for patients with malignant melanoma. Antitumour responses are likely to be mediated by HLA class I- and II-restricted immune responses directed at tumour antigens. We assessed whether the peripheral blood of normal HLA-matched siblings of patients with melanoma could be used to generate lymphocytes with antimelanoma activity for adoptive immunotherapy after allogeneic blood or marrow transplantation. Melanoma cell lines were derived from two donors and were used to stimulate the mononuclear cells of three HLA-identical siblings. CD4^+^ clones dominated cultures. Of these, approximately half were directly cytotoxic towards recipient melanoma cells and secreted interferon-*γ* in response to tumour stimulation. More than half of the noncytotoxic clones also secreted interferon-*γ* after melanoma stimulation. No CD4^+^ clones responded to stimulation with recipient haemopoietic cells. The majority of CD8^+^ clones directly lysed recipient melanoma, but did not persist in long-term culture *in vitro*. No crossreactivity with recipient haemopoietic cells was observed. The antigenic target of one CD4^+^ clone was determined to be an HLA-DR11-restricted MAGE-3 epitope. Antigenic targets of the remaining clones were not elucidated, but appeared to be restricted through a non-HLA-DR class II molecule. We conclude that the blood of allogeneic HLA-matched sibling donors contains melanoma-reactive lymphocyte precursors directed at tumour-associated antigens. Adoptive immunotherapy with unselected or *ex vivo*-stimulated donor lymphocytes after allogeneic stem cell transplantation has a rational basis for the treatment of malignant melanoma.

Allogeneic blood or marrow transplantation is emerging as an effective treatment in some patients with solid tumours, particularly those with renal cell carcinoma (Childs *et al*, 1999; Childs, 2000). Other tumours including breast and ovarian cancers and germ cell tumours may also benefit from this approach (Bay *et al*, 2000, 2002; Pedrazzoli *et al*, 2002; Hanel *et al*, 2003; Donato *et al*, 2004), which is thought to exert its major antitumour effect via donor immune cells active against recipient malignancy (known as the graft-versus-tumour or GVT effect). The GVT effect makes the use of lymphocytes from HLA-identical donors preferable to the use of the patient's own lymphocytes for the purpose of generating cells for immunotherapy.

Melanoma cells express a variety of tumour antigen types that could be the target of immune responses. These include the cancer testis antigens exemplified by the MAGE family as well as differentiation antigens such as tyrosinase and melan-A/MART-1 that are expressed by normal melanocytes as well as melanoma cells (Coulie *et al*, 1993; Boon *et al*, 1994). T-lymphocytes specific for tumour antigens can be detected at low frequency in the circulation of both melanoma sufferers and normal individuals (Lee *et al*, 1999; Valmori *et al*, 2000; Pittet *et al*, 2001). The use of blood from HLA-identical donors offers two major advantages. Patients with melanoma may have deficient immune responses owing to advanced malignancy itself or to the effects of chemoradiotherapy. In addition, the expression of polymorphic tissue antigens by melanoma cells offers additional antigens that can be targeted by allogeneic lymphocytes.

When autologous melanoma-infiltrating lymphocytes expanded *in vitro* are infused into melanoma sufferers following lymphodepleting chemotherapy, a 50% rate of objective response has been observed in patients with metastatic melanoma (Dudley *et al*, 2002, 2005). Tumour targets have included the products of MAGE, HLA, tumour suppressor and other polymorphic genes (Robbins *et al*, 2002; Huang *et al*, 2004; Zhou *et al*, 2005). However, the process of cell generation requires the use of lymphocytes isolated from tumour biopsies and is labour intensive and impractical for routine clinical purposes. To take advantage of the additional alloreactivty provided by polymorphic minor histocompatibility antigens and to utilise the immunocompetent systems of normal donors, we generated melanoma cell lines from two patients with metastatic melanoma and used them to stimulate lymphocytes from the peripheral blood of their HLA-identical siblings. We aimed to determine the ease with which melanoma-reactive clones could be generated and to characterise the clones. We intended to determine the feasibility of infusing *in vitro* expanded melanoma-reactive allogeneic lymphocytes following reduced-intensity lymphodepleting chemotherapy and establishment of allogeneic haemopoietic chimerism in patients with melanoma.

## MATERIALS AND METHODS

### Cell lines

Approval from our Institutional Ethics Committee was granted and informed consent from donors was obtained before collection of any biological samples. Melanoma samples were obtained from surgical biopsies of metastatic lesions in two patients WMPG (tissue type HLA-A1, B44, 49, DRB1 11, 12) and WMMH (tissue type HLA-A3, 24, B7, 35, DRB1 13, 15). Biopsies of melanoma lesions from patients were washed in sterile PBS, cut into small pieces and digested with trypsin-EDTA (Invitrogen, Carlsbad, CA, USA) until all tumour aggregates were disassociated. The cells harvested were centrifuged and cultured in RPMI media+10% FCS at 37°C in a humidified atmosphere containing 5% CO_2_. Nonadherent cells were discarded and adherent cells were passaged using trypsin-EDTA. Cell lines had the appearance of large adherent cells typical of malignant melanoma cells and were characterised by reverse transcriptase–polymerase chain reaction (RT–PCR) for melanoma antigens (see Results). The melanoma lines ET1 and LB33, and the lymphoblastoid cell line (LCL) LG2-EBV were kindly provided by Professor T Boon (Ludwig Institute for Cancer Research, Brussels, Belgium). The melanoma lines MD-TC, OI-TC, GRA and the HLA-DR11+ BOR-EBV were a kind gift of Dr MP Protti (Scientific Institute H San Raffaele, Milan, Italy). Me18732 was kindly provided by Dr Parmiani (Istituto Nazionale dei Tumori, Milan, Italy). Peripheral blood mononuclear cells (PBMCs) were isolated from patients or their HLA-A-, -B-, -DRB1-identical donors by Lymphoprep (Nycomed, Oslo, Norway) gradients. Activated T cells were obtained by cultivation of PBMC, in the presence of 1 *μ*g ml^−1^ of PHA (Boehringer Mannheim, Mannheim, Germany) and 100 U ml^−1^ r-hu-IL-2 (Chiron, Milan, Italy). B-lymphoblastoid cell lines were derived by transformation of peripheral blood B-lymphocytes with the B95-8 strain of EBV, obtained from the Department of Virology, Institute for Clinical Pathology and Medical Research, Westmead Hospital. Bone marrow fibroblasts were isolated by adherence from marrow aspirates and were cultured in RPMI+10% FCS, passaged at confluency and reseeded at 10% confluency.

### Flow cytometry

Antibodies to human CD3, CD4, CD8, CD13, CD19, CD56, CD80, CD86, CD62L, interferon-*γ* and IL-4 were purchased from BD Biosciences (San Jose, CA, USA). W6/32 (anti-HLA class I) was purchased from Sigma-Aldrich (St Louis, MO, USA). Flow cytometry was carried out on a FACSCalibur flow cytometer (BD Biosciences, San Jose, CA, USA) and analysed using CellQuest software. To label intracellular antigens including cytokines, cells were first fixed by incubation in 4% paraformadehyde. After washing, cells were incubated in ICPerm (Biosource, Nivelles, Belgium) and were labelled with primary antibodies and washed in PBS. If the primary antibody was not conjugated to fluorochromes, fluorochrome-conjugated secondary antibody was added and cells were incubated before washing and analysing on the flow cytometer.

### *In vitro* induction of tumour-specific effectors

Autologous mixed lymphocyte tumour cell cultures (MLTCs) were established as described previously (Van den Eynde *et al*, 1989) using irradiated autologous tumour in the presence of 10 U ml^−1^ IL-2 (Chiron, Milan, Italy) and 5 ng ml^−1^ IL-7 (Genzyme Corp., Cambridge, MA, USA). Cultures were maintained in IMDM+10% pooled human AB serum supplemented with 0.55 mM L-arginine, 0.24 mM L-asparagine and 1.5 mM L-glutamine, 50 U ml^−1^ penicillin and 50 *μ*g ml^−1^ streptomycin. Lymphocytes were stimulated weekly and tested in cytotoxicity or cytokine release assays after three stimulations. On day 21, lymphocytes from the culture were cloned by limiting dilution and maintained in culture as described previously (Traversari *et al*, 1989). In brief, lymphocytes from MLTCs were counted and seeded in 96-well plates at a ratio of 0.3 cells well^−1^. To each well, 500 melanoma cells (*γ*-irradiated to 90 Gy), 30 000 EBV-transformed feeder lymphoblastoid cells (irradiated to 90 Gy) and IL-2 (final concentration 50 U ml^−1^) were added in a total culture volume of 200 *μ*l well^−1^. Half of the media and IL-2 were replaced every 3–4 days, and fresh irradiated melanoma cells and EBV-LCL feeder cells were added weekly as above. After 3–4 weeks, wells were examined by light microscopy for outgrowth of lymphocytes. Positive wells were maintained at 10^5^ cells well^−1^ until expanded sufficiently for testing.

### Cytotoxicity assay

The lytic activity of CTLs was tested in a classical chromium release assay (Gottlieb *et al*, 1989), in the presence of 30 : 1 excess of cold K562 cells. For blocking assays, the effector cells were preincubated with Ab specific for CD3 (OKT3: Orthoclone, Milan, Italy), CD4, CD8 or alternatively target cells were preincubated with anti-MHC class I (W6/32) and anti-HLA-DR (L243) Abs 30 min at room temperature. Effector and target cells were added in 96-well microplates at a fixed E : T ratio to evaluate the lytic activity as ^51^Cr release after 4 h.

### Cytokine secretion assay

Responder cells (1 × 10^5^) at day 5 after stimulation and stimulator cells (3 × 10^5^) were mixed in 150 *μ*l of IMDM 10% human serum supplemented with 25 U ml^−1^ IL-2. After 24 h at 37°C, 100 *μ*l of supernatant were harvested, and interferon-*γ* concentration was measured using an interferon-*γ* ELISA kit (BD Biosciences Pharmingen, San Diego, CA, USA) according to the manufacturer's recommendations. Blocking experiments were performed as described above.

## RESULTS

### Characterisation of melanoma cell lines

**Figure 1:**
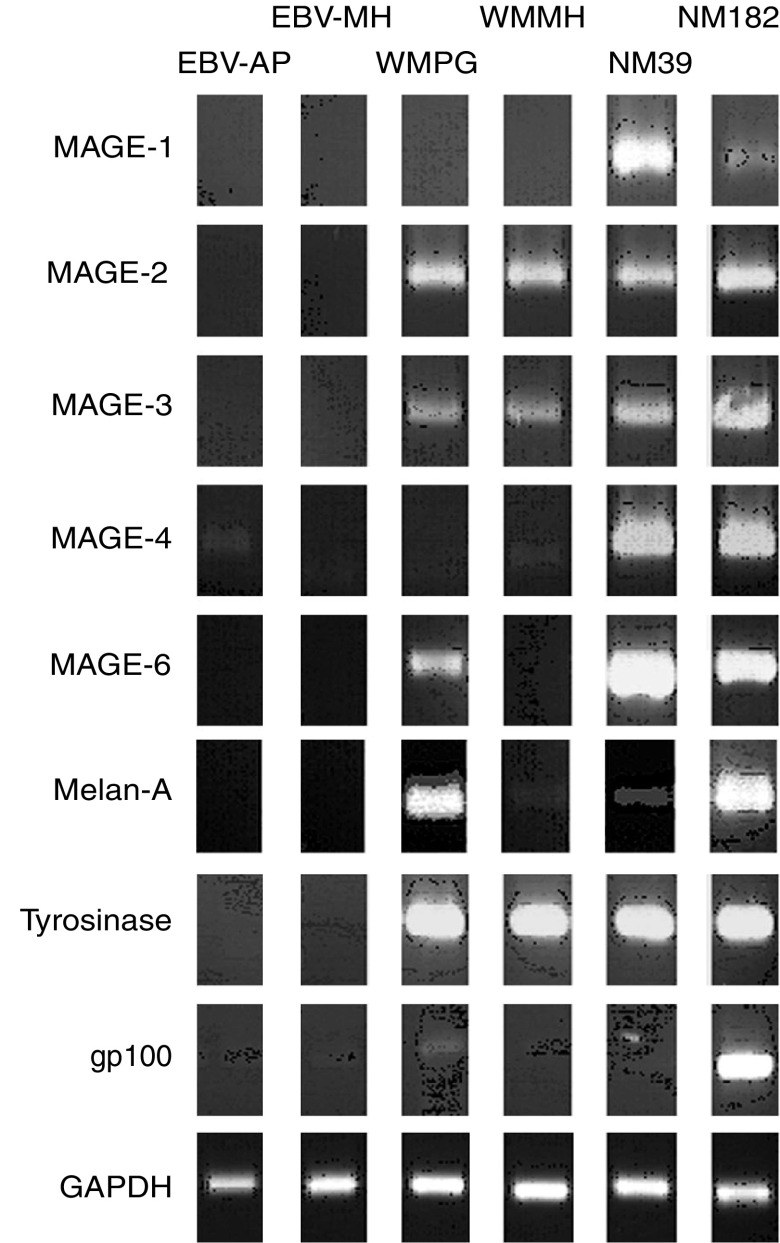
Expression of melanoma antigens by WMPG and WMMH. Reverse transcriptase–polymerase chain reaction was used to assess expression of a panel of common melanoma antigens by WMPG and WMMH. RNA was extracted from WMPG and WMMH, reverse-transcribed and amplified with primers that anneal to mRNAs of melanoma antigens. Reverse transcriptase–polymerase chain reaction products of two EBV-LCL cell lines (EBV-AP and EBV-MH) and two other melanoma cell lines (NM39 and NM182) were included as negative and positive controls, respectively. Amplification of GAPDH was shown as loading control. Bands of sizes corresponding to amplification of mRNAs (instead of genomic DNA) were shown.

**Figure 2:**
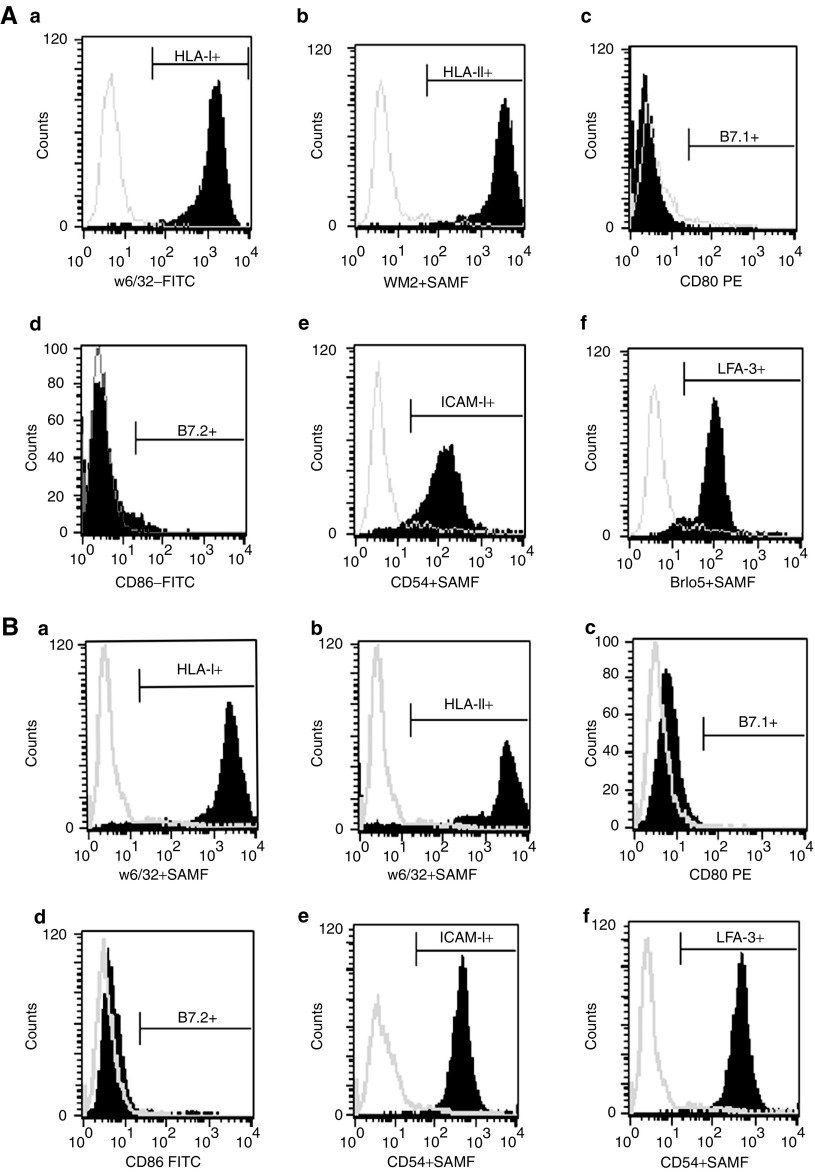
Expression of HLA, costimulatory molecules and adhesion molecules by WMPG (**A**) and WMMH (**B**). Cells were stained with antibodies specific for (a): HLA class I; (b): HLA class II; (c): B7.1; (d): B7.2; (e): ICAM-1 and (f): LFA-3 and assessed by flow cytometry. Grey lines represent staining with isotype control antibodies, and black lines staining with the indicated antibodies.

Cell lines WMPG and WMMH grew as adherent cells with the typical morphological appearance of melanoma cells. By PCR, WMPG was shown to express MAGE-2, MAGE-3, MAGE-6, Melan-A, tyrosinase and gp100, whereas WMMH expressed MAGE-2, MAGE-3, tyrosinase and Melan-A (Figure 1). Both WMPG and WMMH expressed high levels of HLA class I and II, ICAM-1 and LFA-3 (Figure 2). However, neither cell line expressed B7.1 or B7.2, the principal costimulatory molecules required for the activation of naïve T cells (Figure 2). Additionally, neither cell line expressed cell surface antigens of T, B or natural killer origin (data not shown).

### Generation and characterisation of T-cell clones

Mixed lymphocyte tumour cell cultures were established using WMPG melanoma cells and effectors from his HLA-identical sibling, and from WMMH and effectors from each of two HLA-identical siblings. After limiting dilution cloning, a total of 49 lymphocyte clones was obtained from 1560 seeded wells. Only two out of a total of 49 clones were CD8^+^, the remainder being CD4^+^. To derive CD8^+^ T-cell clones for further study, MLTCs were sorted into >99% pure CD4^+^ and CD8^+^ fractions and CD8^+^ T cells were then cloned by limiting dilution. From a total of 960 seeded wells, 47 CD4^+^ and 65 CD8^+^ clones were obtained (Table 1). The phenotype of clones was verified periodically throughout this study. The CD8^+^ clones expanded during the limiting dilution process, but none was able to persist and expand in culture for more than 2 months after limiting dilution despite coculture with OKT3 or irradiated allogeneic PBMC from healthy donors or EBV-transformed feeder cells (LG2-EBV). This limited their functional characterisation. On the other hand, a number of CD4^+^ T-cell clones were able to survive and expand for greater than 4 months in culture, enabling their further characterisation in subsequent experiments.

**Figure 3:**
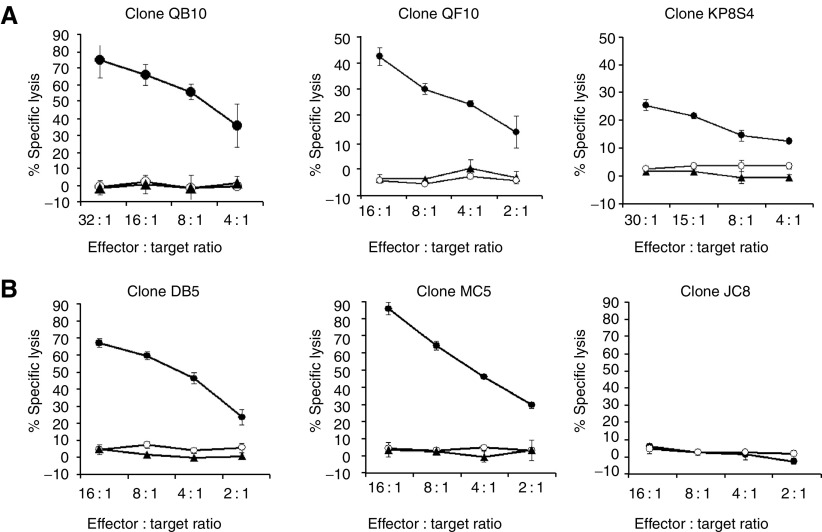
Cytotoxicity of T-cell clones. CD8^+^ T-cell clones against recipient melanoma cells. (**A**) CD8^+^ T-cell clones QB10 and QF10 from the WMPG donor–recipient pair, and clone KP8S4 from the WMMH donor–recipient pair were used as effectors against recipient melanoma cells (solid dots), the feeder cells LG2-EBV (open circles) and cell lines of recipient origin (PHA-PG for QB10 and QF10, EBV-MH for KP8S4; solid triangles). Error bars represent 95% confidence interval (CI) calculated from triplicates. (**B**) CD4^+^ T-cell clones DB5, MC5 and JC8 were used as effectors in cytotoxicity assays against WMPG (solid dots), the feeder cells LG2-EBV (open circles) and PHA blasts from WMPG (solid triangles). Error bars represent 95% CIs calculated from triplicates.

For a number of CD8^+^ T-cell clones, cytotoxicity against recipient melanoma cells was demonstrated in ^51^Cr-release cytotoxicity assays. Data from representative clones are shown in Figure 3A. Clones QB10 and QF10 were generated by limiting dilution from CD8^+^-sorted MLTC of the WMPG donor/stimulator pair. Clone KP8S4 was derived by limiting dilution from CD8^+^-sorted MLTC of the WMMH donor/stimulator pair. Clones QB10 and QF10 killed recipient melanoma WMPG cells but did not show cytotoxicity against LG2-EBV feeder cells nor against PHA blasts derived from the originator of the WMPG line, suggesting that clones QB10 and QF10 recognised an antigen expressed by melanoma rather than a minor histocompatibility antigen expressed by tumour cells. This pattern of cytotoxicity was observed in 38 out of 45 CD8^+^ T-cell clones raised from the donor against WMPG. Seven out of 45 CD8^+^ clones showed no cytotoxicity against either WMPG or EBV feeder cells. As shown in Figure 3, KP8S4 killed recipient melanoma WMMH but not LG2-EBV feeder cells nor EBV-transformed cells from the originator of the WMMH line, suggesting that KP8S4 also recognised an antigen expressed by WMMH melanoma but not a minor histocompatibility antigen expressed by donor MH. Only two CD8^+^ T-cell clones derived from WMMH-stimulated cultures (including KP8S4) could be expanded for testing. As the aim of the study was only to analyse the characteristics of clones generated from HLA-identical siblings, no attempt was made to expand clones to numbers that might be required for therapeutic purposes.

**Figure 4:**
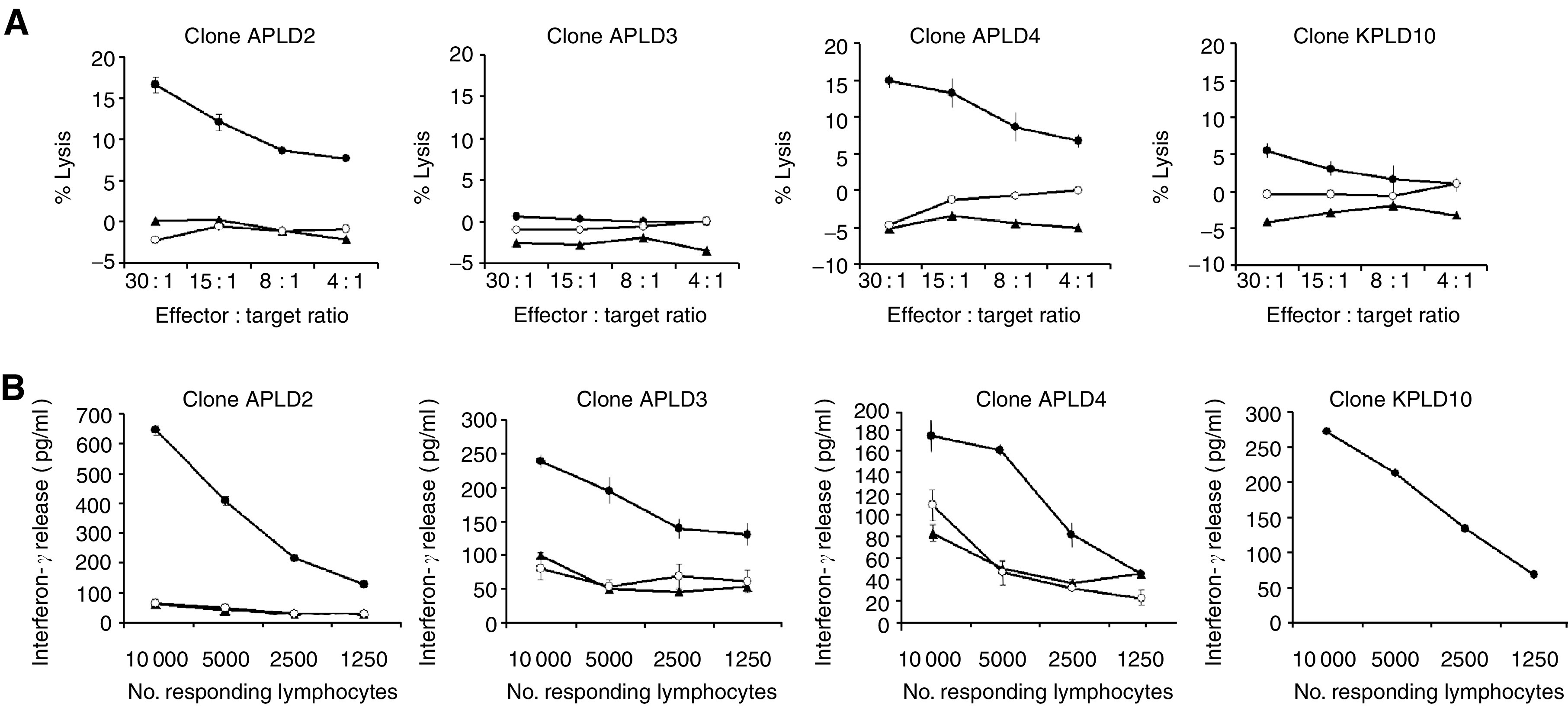
(**A**) Cytotoxicity of CD4^+^ clones raised from WMMH donors. Clones APLD2, APLD3, APLD4 and KPLD10 were used as effectors against recipient melanoma (solid dots), recipient-derived EBV-LCL (solid triangles) and the feeder cells LG2-EBV (open circles) in cytotoxicity assays. Error bars represent 95% CI calculated from triplicates. (**B**) Interferon-*γ* release by CD4^+^ clones raised from WMMH donors. Clones APLD2, APLD3, APLD4 and KPLD10 from donors AP and KP were used as effectors against targets consisting of recipient melanoma WMMH (solid dots), recipient-derived EBV-LCL (EBV-MH; solid triangles) and the feeder cells LG2-EBV in a cytokine secretion assay. Error bars represent 95% CI calculated from triplicates. Results were representative of at least two experiments.

In contrast to CD8^+^ clones, most CD4^+^ T-cell clones were able to survive and expand in culture, enabling their further characterisation. Recognition of WMPG by the lymphocytes was assessed in ^51^Cr-release cytotoxicity assays. Results from three representative clones are shown in Figure 3B. CD4^+^ clones DB5 and MC5 killed the recipient melanoma cell line WMPG specifically and not recipient PHA blasts or the LG2-EBV feeder cell line. However, another clone JC8 failed to lyse the original WMPG. Of the 14 CD4^+^ clones characterised, four clones displayed cytotoxicity patterns similar to clones DB5 and MC5. Another three clones killed WMPG, but cytotoxicity assays were not performed against PHA blasts owing to the clones dying in culture. The remaining seven CD4^+^ clones did not kill WMPG. A total of 25 CD4^+^ T-cell clones were derived from the two HLA-identical siblings of the WMMH line. These clones were tested against WMMH targets in ^51^Cr-release cytotoxicity assays (Figure 4A). Two patterns of cytotoxicity were observed. Half of the CD4^+^ clones derived exhibited cytotoxicity directly against recipient melanoma cells without killing a WMMH-derived EBV cell line, implying the clones did not recognise a minor histocompatibility antigen. The remaining clones did not kill the WMMH melanoma line or EBV-transformed recipient or feeder cells. All clones that were cytotoxic secreted interferon-*γ* in response to stimulation with WMMH (Figure 4B). However, four of the six noncytotoxic clones also released interferon-*γ* in response to WMMH. No clones tested secreted interferon-*γ* in response to stimulation with WMMH EBV-transformed cells or LG2-EBV feeder cells, indicating that no clones recognised minor histocompatibility antigens expressed by the tumour cells or the patient's EBV-transformed cells.

### Characterisation of target epitopes of CD4^+^ T-cell clones

**Figure 5:**
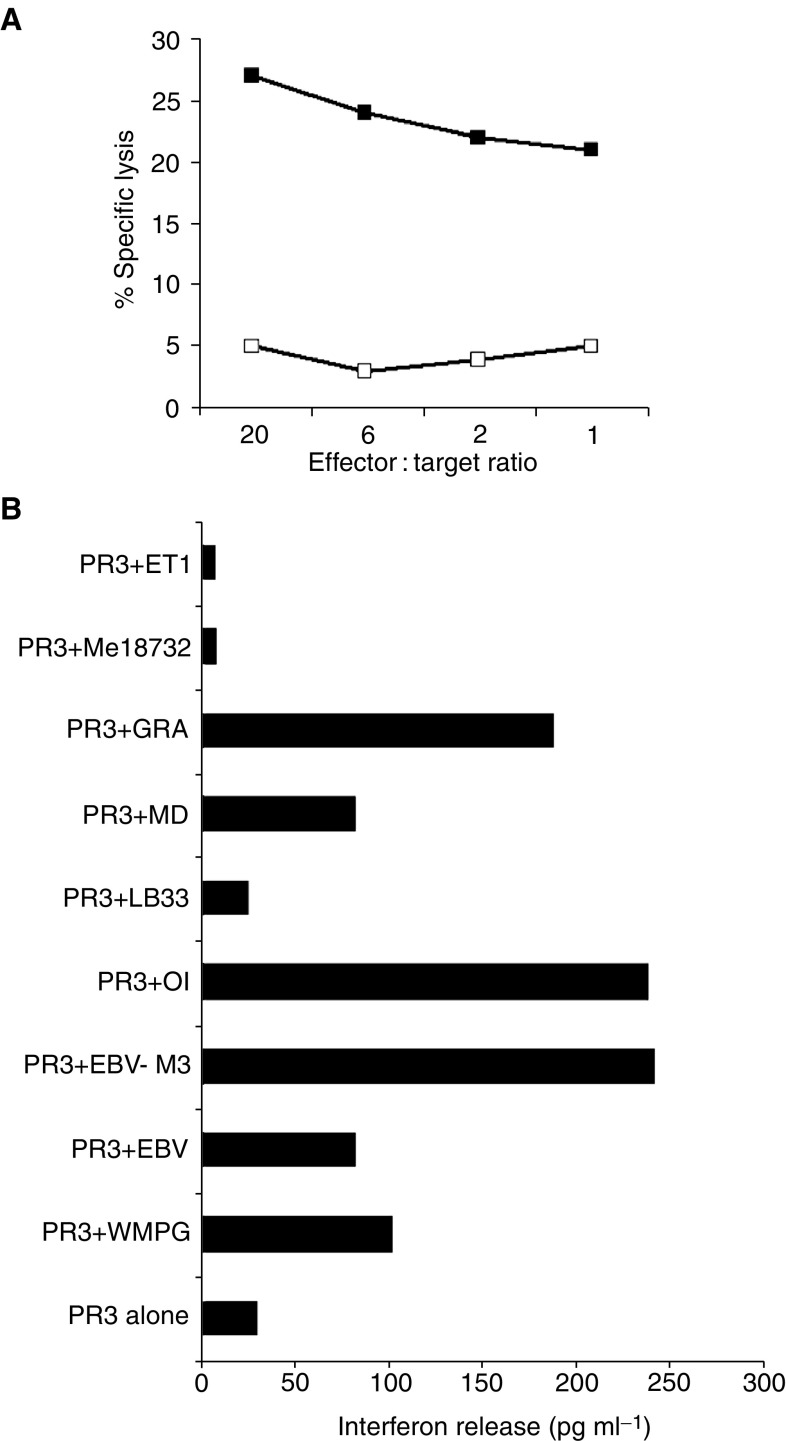
Characterisation of clone PR3. (**A**) Specific lysis by clone PR3 against an MAGE-3-transfected (closed squares) or a vector-only-transfected (open squares) HLA-DR11+ EBV-transformed cell line. (**B**) Interferon-*γ* release by clone PR3. Interferon-*γ* release by PR3 cells was measured following culture with HLA-DR11− melanoma cells (ET-1, Me18732), HLA DR11+ melanoma cells (GRA, MD, LB33, OI and WMPG) and an HLA DR11+ EBV-transformed cell line either control-transfected (EBV) or transfected with MAGE-3 (EBV-M3). Interferon-*γ* release by the stimulating cells was subtracted.

**Figure 6:**
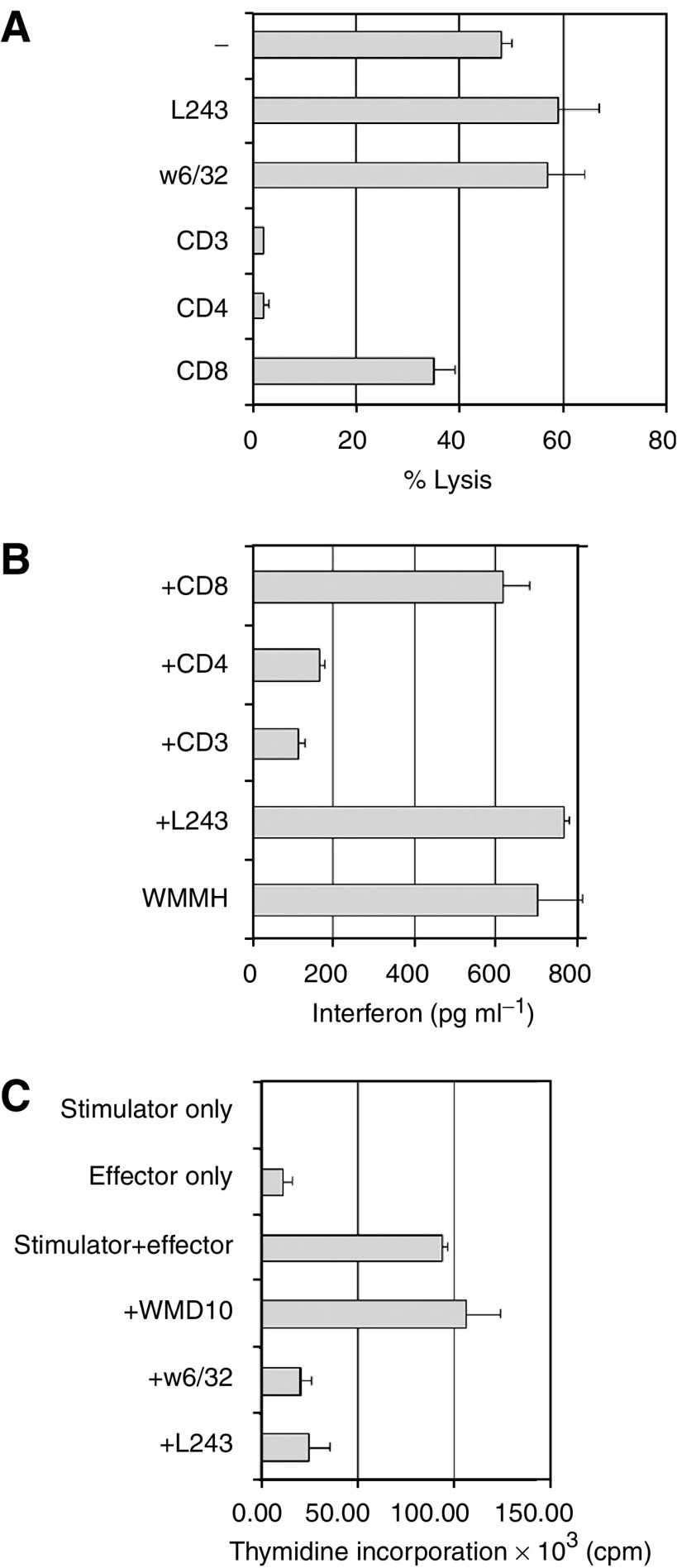
Inhibition of APLD2 cytotoxicity and interferon- release. (**A**) L243, W6/32, OKT3, anti-CD4 and anti-CD8 Abs were added to 4-h ^51^Cr-release cytotoxicity assays using APLD2 as effector and WMMH as targets. (**B**) The same Abs were also used to block overnight interferon- release by APLD2 against WMMH. (**C**) To test the validity of the Ab preparations, the Abs were used to block 5-day one-way mixed lymphocyte reactions using PBMCs from random allogeneic normal donors. Cytotoxicity assays were performed in triplicates, and error bars represent 95% CI of triplicates. Percentage-specific lysis at an effector : target ratio of 50 : 1 is shown. Dose–responses using lower effector : target ratios were observed and are not shown. Interferon-*γ* release assay was performed in duplicate and error bars represent range of duplicates. ^3^H-thymidine incorporation assay to measure lymphocyte proliferation was performed in quadruplicates and error bars represent 95% CIs. All results representative of two experiments.

An attempt was made to identify the antigen(s) recognised by CD4^+^ clones. MAGE-3-derived peptides presented by HLA-DR11 and recognised by CD4^+^ T-lymphocytes have previously been identified (Hahn *et al*, 1995). To test if any of the clones generated from the HLA-identical donor of the WMPG patient (that expressed HLA-DR11) recognised MAGE-3 peptides in association with HLA-DR11, CD4^+^ clones were used as effectors in cytotoxicity assays against an HLA-DR11+ EBV-LCL engineered to express MAGE-3. One of seven clones tested (clone PR3) was cytotoxic against the MAGE-3 transfectant, but not the vector-only-transfected line (Figure 5A). To validate this result, clone PR3 was tested for its ability to secrete interferon-*γ* in response to stimulation with six melanoma cell lines (four HLA-DR11+, two HLA-DR11−). Clone PR3 secreted interferon-*γ* in response to WMPG, the MAGE-3-transfected HLA-DR11+ EBV-transformed cell line and three of four HLA-DR11+ melanoma cell lines, but did not secrete interferon-*γ* after stimulation with either of the HLA-DR11− melanoma lines or after stimulation with an HLA-DR11+ line (LB33) with low HLA-DR expression (Figure 5B and data not shown). The remaining clones failed to respond to either MAGE-3-transduced or -untransduced HLA-DR11+ EBV lines. CD4^+^ clone APLD2 isolated from one of the HLA-identical donors of the originator of the WMMH line killed the WMMH cell line but not EBV-transformed cell lines from the WMMH donor (see above). An attempt was made to identify the class II restriction element by inhibiting the cytotoxicity with the anti-HLA-DR monoclonal antibody L243. The activity of the APLD2 clone was inhibited by anti-CD3 and anti-CD4 Abs; however, no inhibition of cytotoxicity or interferon-*γ* release could be obtained implying that the restriction element must be another (non-HLA-DR) class II determinant (Figure 6). A donor EBV-transformed cell line was generated and transfected with retroviral constructs containing MAGE-2, MAGE-3 or Melan-A. None of the eight clones tested secreted interferon-*γ* against any of the melanoma antigen-transfected cell lines (data not shown).

## DISCUSSION

Infusion of autologous *in vitro* expanded tumour-infiltrating lymphocytes into lymphodepleted hosts suffering from melanoma is associated with high rates of disease response in patients with advanced disease (Dudley *et al*, 2005). The use of allogeneic donors as a source of immune system cells could have benefits, in particular the recognition of minor histocompatibility antigens on tumour cells by allogeneic donor lymphocytes and the potential for improved functional immune responses using cells from noncompromised healthy donors. In this work, we assessed the potential for HLA-A-, -B- and -DR-identical sibling donors to act as a source of melanoma-reactive lymphocytes that could exert a GVT effect if infused after allogeneic stem cell transplant. Both CD4^+^ and CD8^+^ clones, particularly the former, could be generated from the blood of HLA-identical normal sibling donors of melanoma sufferers. All previous reports of melanoma-reactive cytotoxic cells raised from normal donors, including the only other study using HLA-matched identical donors (Kurokawa *et al*, 2002), have identified dominant MHC class I-restricted responses in cultured cells. (Kurokawa *et al*, 2002; Glazyrin *et al*, 2003; Nolte *et al*, 2003). Our report is the first to demonstrate the generation of predominantly CD4^+^ melanoma-reactive clones, many interferon-*γ* secreting, from normal donors.

We generated cell lines from biopsies of metastatic lesions of two patients with melanoma. These lines had the morphological and culture features of neoplastic cells and expressed antigens typical of melanoma cells. Using PBL of HLA-identical siblings as responder cells, melanoma-reactive effectors were isolated from three HLA-identical siblings of the two melanoma cell line originators. Clones were obtained from MLTCs with an overall cloning efficiency of approximately 3%. Both CD4^+^ and CD8^+^ clones were melanoma reactive, but CD4^+^ clones dominated the cultures. CD8^+^ clones derived after cell sorting of bulk cultures failed to persist for longer than 2 months *in vitro*. Despite this, some CD8^+^ clones efficiently lysed parental melanoma lines. In cytokine release assays, CD8^+^ clones released interferon-*γ* but not IL-4 (data not shown) in response to stimulation with melanoma lines consistent with the acquisition of a Tc1 phenotype.

The reason for the dominance of CD4^+^ clones and the failure to propagate long-term CD8^+^ clones from these cultures is unclear. Possibilities include the specific antigen stimulus (Glazyrin *et al*, 2003), our use of whole tumour cells and the class I and II HLA matching in this allogeneic system. Although the original experiments cloning melanoma-reactive CD8^+^ CTL were also performed with whole autologous tumour cells (Herin *et al*, 1987), others have also reported difficulty isolating CD8^+^ CTL clones following this type of stimulation (Takahashi *et al*, 1995). In one study that generated tumour-specific CD8^+^ cytotoxic T cells from allogeneic donors, MLTCs were initiated with a highly purified population of CD8^+^ donor lymphocytes potentially underestimating the role of CD4^+^ clones in the immune response. (Dorrschuck *et al*, 2004). Our data are the first to document the results of cloning MHC class I and II HLA-matched allogeneic effector cells stimulated with whole tumour cells and are in line with previous observations made using autologous MLTC (Takahashi *et al*, 1995).

Approximately half of the CD4^+^ clones were weakly cytotoxic against the melanoma cell line used for stimulation. More than half of the remaining noncytotoxic CD4^+^ clones released interferon-*γ* on exposure to the melanoma line. Several other studies have reported isolation of HLA class II-restricted melanoma-specific CD4^+^ CTLs, including clones recognising MAGE-3, NY-ESO1 and tyrosinase epitopes (Kobayashi *et al*, 1998; Chaux *et al*, 1999; Manici *et al*, 1999; Zarour *et al*, 2000). We isolated a CD4^+^ HLA-DR11-restricted clone directed against an MAGE-3 epitope from one of our three normal donors. We found no evidence of class I restriction in any CD4^+^ clone as has been described previously (LeMay *et al*, 1993; Mitchell *et al*, 1993; Darrow *et al*, 1996). Restriction of MAGE epitopes via non-HLA-DR class II molecules as described previously is a more likely explanation for our findings (Schultz *et al*, 2000; Mandic *et al*, 2005). None of the CD4^+^ T-cell clones established in this study secreted interferon-*γ* in response to recipient-derived nonmelanoma cells. Similarly, CD8^+^ clones did not lyse normal cells of melanoma patients, suggesting that sibling donor cells capable of mediating graft-versus-host reactions did not dominate the cultures. Although in most cases the target antigen could not be identified, the failure to lyse normal patient cells suggested that melanoma-specific or melanoma-associated rather than minor histocompatibility antigens were common targets of clones isolated from normal donors.

Our data do not allow us to assess the use of HLA-identical sibling donor lymphocytes as a starting population for the generation of antimelanoma cells for adoptive therapy. Our CD4^+^ clones proliferated well, but we did not specifically address the question of expansion to therapeutic numbers beyond 10^9^ cells in total. Further testing is needed before it can be determined whether a sufficient number of melanoma-reactive cells can be obtained for therapeutic purposes from HLA-identical donors of melanoma sufferers. Nevertheless, our data allow us to conclude that melanoma-reactive precursor cells exist in the peripheral blood of such donors and that the precursors of melanoma-reactive CD4^+^ T cells are common. Although we cannot be certain of the role of CD4^+^ cells in mediating antimelanoma tumour immunity, they may have a number of effects: direct antitumour effector cells, provision of help to CD8^+^ clones with greater direct cytotoxic potential or assistance in the generation of lymphocytes with long-lived memory. They have beneficial therapeutic effects in animal models even when not directly lytic *in vitro* (Kahn *et al*, 1991; Shinomiya *et al*, 1995). If, as described in cytomegalovirus infection (Gamadia *et al*, 2003), the presence of interferon-*γ*-secreting CD4^+^ cells is essential for the development of mature and effective CD8 and B-cell responses, infusion of an mixed population of melanoma-reactive cells of both CD4^+^ and CD8^+^ phenotype may assist CD8^+^-cell survival and maturation and result in enhancement of a broad antimelanoma immune response.

## Figures and Tables

**Table 1:** Number of CD4+ and CD8+ clones derived from (a) WMPG-JG donor pair, (b) WMMH-AP donor pair and (c) WMMH-KP donor pair

	**CD4+ (recognising stimulator)**	**CD8+ (recognising stimulator)**
(a) WMPG-JG	22 (7/10/5)	45 (38/6/1)
(b) WMMH-AP	13 (6/1/6)	6 (0/0/6)
(c) WMMH-KP	12 (5/1/6)	14 (2/0/12)

Recognition of stimulator cell was determined either by Cr-release cytotoxicity assay or interferon-*γ* release as described in Materials and Methods. Numbers in brackets show the number of clones recognising, not recognising and not tested against the original stimulator cells, respectively.
